# Ethical challenges assessed in the clinical ethics Committee of Psychiatry in the region of Southern Denmark in 2010–2015: a qualitative content analyses

**DOI:** 10.1186/s12910-018-0308-z

**Published:** 2018-06-19

**Authors:** H. Bruun, S. G. Lystbaek, E. Stenager, L. Huniche, R. Pedersen

**Affiliations:** 1 Department of Regional Health Research, University of Southern Denmark, Odense, Denmark; 2 Psychiatric hospitals, the Region of Southern Denmark, Toldbodgade 45, 5000 Odense, Denmark; 3 User Perspectives, Department of Public Health, University of Southern Denmark, Odense, Denmark; 4 Center for Medical Ethics, Institute of Health and Society, University of Oslo, Oslo, Norway

**Keywords:** Clinical ethics committee, Mental healthcare, Ethical challenges, Health-care professionals, Qualitative content analysis

## Abstract

**Background:**

The aim of this article is to give more insight into what ethical challenges clinicians in mental healthcare experience and discuss with a Clinical Ethics Committee in psychiatry in the Region of Southern Denmark. Ethical considerations are an important part of the daily decision-making processes and thereby for the quality of care in mental healthcare. However, such ethical challenges have been given little systematic attention – both in research and in practices.

**Methods:**

A qualitative content analysis of 55 written case-reports from the Clinical Ethics Committee. The Committee offers clinicians in mental healthcare structured ethical analyses of ethical challenges and makes a thorough written case-report.

**Results:**

The ethical challenges are grouped into three overarching topics: 1. Clinicians and their relation to patients and relatives. 2. Clinicians and institutional aspects of mental healthcare 3. Clinicians and mental healthcare in a wider social context. Through presentation of illustrative examples the complexity of daily clinical life in mental healthcare becomes evident, as well as typical interests, values and arguments.

**Conclusions:**

This qualitative study indicates that difficult ethical challenges are an inherent part of mental healthcare that requires time, space and competence to be dealt with adequately.

## Background

The awareness that ethical considerations form an inseparable part of mental healthcare, has led to the creation of various ethics support services in different countries. Important examples are clinical ethics committees [1], moral case deliberation [2, 3], ethical reflection groups [4] and ethics consultants.

The purpose of this article is to give more insight into what ethical challenges clinicians in mental healthcare experience and discuss with a Clinical Ethics Committee in psychiatry. Furthermore, through the presentation of illustrative case-examples we want to describe typical interests, values and arguments inherent in these ethical challenges. There are several reasons why this is important. Firstly, dealing with ethical questions is an important part of the daily decision-making processes and quality of care in mental healthcare. Secondly, deliberations on ethical issues in mental healthcare tend to be theoretical rather than based upon empirical data. Thirdly, there is little knowledge about the ethical challenges experienced by clinicians in mental healthcare more broadly and not just in relation to the use of coercion. Such knowledge is important to those engaged in mental healthcare: members of ethical committees, those responsible for education, managers and healthcare professionals. Furthermore, this knowledge is important for patients, relatives and the general public because it brings to notice important and difficult ethical challenges in mental health services.

The point of departure for ethics support services varies between countries and healthcare institutions [5]; for instance as to how the services are organised and used by healthcare professionals, and their status and function in the healthcare organisations. That is the case even in the Scandinavian countries, where the overall structure of the healthcare systems is very similar. For example, unlike its neighboring country Norway, in Denmark there is no national requirement demanding hospital trusts to secure clinicians access to ethical consultant services. In general, Denmark has no long tradition for providing ethical consultation services in hospital settings; the first Danish clinical ethics committee for a somatic hospital was established as a local initiative in 2004. The Region of Southern Denmark established its first Clinical Ethics Committee of Psychiatry in 2010; later an additional four Committees have been established to assist clinicians in somatic healthcare. By contrast, Norway established its first three Clinical Ethics Committees as early as 1996; they are used mainly by somatic clinicians, but also by clinicians working in mental healthcare [6].

Mental healthcare in Denmark is an integrated part of the overall public healthcare system, which is funded by the government. All patients suffering from mental illness have equal right to treatment. Psychiatrists are responsible for the treatment, which is provided in cooperation with clinicians with interdisciplinary backgrounds. Mental healthcare is regulated by Danish health legislation and clinical guidelines issued by the Danish Health Authority. On a national level, health legislation describes the foundation of medical treatment as informed consent and the duty of clinicians to protect patient confidentiality. However, health legislation also contains instructions about breaking the duty of confidentiality if it is deemed necessary to protect patients and others from foreseeable harm. In Danish mental healthcare there is a strong focus on safeguarding the patient’s right of self-determination and on reducing the use of coercion. In some situations, however, coercion is seen as the least intrusive way to protect the patients from additional deterioration of their illness, or to avoid harm either to the patients themselves or others. The use coercion in Danish psychiatry is regulated in the Mental Health Act. To be subject to this Act, a patient must be mentally ill and psychotic. In Denmark, clinicians such as doctors, nurses and psychologists have specific professionally relevant codes of ethics guiding their work, but there is no specific ethical code for psychiatrists.

It is in this context that the Clinical Ethics Committee of Psychiatry in the Region of Southern Denmark is offering deliberation on ethical challenges experienced by clinicians in their daily clinical life. The purpose is not to prevent court cases or to offer specific resolutions, but to contribute towards mutual learning when a case is elucidated. Often clinicians can be inspired to find alternative ways of action in future similar situations. From a Norwegian evaluating study [7] we know that clinicians find the work of such committees useful and worthwhile. One reason is that all pros and cons of the case are looked into; another is the systematic and thorough analysis of the ethical challenge. The clinicians state that their concerns are treated seriously, and that the privatisation of ethical challenges is reduced. The clinicians find that the systematic discussions positively influence how subsequent problems are dealt with in their departments. One clinician states that consulting the Committee has helped staff see the patients’ wishes and values more clearly. Others mention that the discussions have given the patients’ relatives a feeling of being taken more seriously. In the same paper important obstacles, for referring a case to a committee is described. Clinicians experience that within the medical culture there is a tendency to evade conflict and outside involvement. Also, there is a risk of the committee being described by clinicians as a moral court.

To overcome this obstacle, the Committee of Psychiatry in the Region of Southern Denmark has stated very explicitly that the purpose of the Committee is not that of a moral court. Instead, it is to support clinicians in their decision-making processes and to promote the quality of ethical reflections in order to make actions more well-grounded, and to elicit and include the perspectives of patients as well as clinicians.

Available research [6, 8], albeit limited, public debates, and the authors’ own experiences indicate that ethical challenges in mental healthcare are both complex and frequent, while ethics support services are less developed than in other fields of healthcare [9]. One example of research is Reiter-Theil et al. [10] using the “Encyclopedia of Bioethics” to classify the ethical content of 50 ethics consultations performed at the Psychiatric Hospital of the University Basel. The most frequent ethical content in the 50 cases included: coercion (17:50), care-management (10:50) and treatment-plan evaluation (10:50). In addition, there are various studies focusing on special fields or topics within mental healthcare, e.g. coercion [11, 12] or child and adolescent psychiatric in-patient care [13]. In somatic healthcare and community care, cases dealt with in ethics support services have been studied more extensively to describe real-life ethical challenges [14–16].

### The clinical ethics Committee in Psychiatry in the region of Southern Denmark

The members of the Committee are doctors, nurses and other clinicians such as psychologists, nursing assistants, social workers and occupational therapists, all of whom are in daily contact with patients. One or two of the Committee members have managerial responsibilities in wards at psychiatric hospitals. The members are appointed for a period of 2 years, which can be prolonged for a further 2 years. In addition to the clinicians, a lawyer, a priest and a philosopher are members, as are a patient and a relative representative. The Committee uses a wide definition of what an ethical challenge is: “An ethical challenge arises when there is doubt, uncertainty or disagreement about what is right or good” [12].

The Committee receives cases from the entire Region of Southern Denmark, which has six mental hospitals for adult psychiatry and three mental hospitals for child and adolescent psychiatry. All nine hospitals have in- and outpatient clinics. Ethical challenges submitted to the Committee are analysed in a systematic way. A modification of the “SME model” (a deliberation model developed at Centre for Medical Ethics at the University of Oslo) [1] is used. The SME model presents the following questions: What is the ethical dilemma? What are the facts in the case? Who are the parties involved? What do the relevant ethical positions, values and norms say about the dilemma? What laws and guidelines are relevant? What are the possible ways of action? The purpose of this systematic and structured ethical analysis is to elevate the deliberations from an intuitive level to a critical-evaluative level [17]. This is done by introducing professional codes of ethics, ethical rules and ethical principles. To assist the members of the Committee, supportive questions have been added to each main question. This is done to secure a thorough discussion on a variety of ethical aspects. Table 1 shows the supportive questions.

**Table 1:** Modified “SME model” with supportive questions

What is the ethical dilemma?	What are the facts in this case and what laws and guidelines are relevant?	Who are the involved parties?	What does the relevant ethical positions, values and norms say about the dilemma?	What are the possible ways of action?
Specify, what makes the situation difficult? Clarify, what is at stake and what the ethical challenge is?	What relevant information is available? Is relevant information missing? What laws and guidelines are relevant? Are there any special elements to consider? Is the ethical challenge well-known in clinical practice? What is the usual way of handling the ethical challenge? What is the treatment plan and what is it the intention to obtain with it?	Who are affected? Who can/must make a choice? Identify – if possible – what the involved parties knows and what their values, wishes and intentions are. What consequences does the outcome have for the involved parties? Can we build up or maintain a relationship of trust? How is this ethical challenges related to the core values of Danish psychiatry: respect, professional competency and responsibility?	Autonomy • Has the patient been informed and asked? • Is the patient in a state where he can evaluate the consequence of his choice? Is the patient consistent? • Is the patient under pressure? What would be to the best of the patient? • How are the wishes and values of the patient respected? • How is integrity, dignity, vulnerability of the patient respected? • How is the level of confidence? How do we avoid causing harm? • Does the good we do outweigh the potential harm - to the patient, to others affected, and to the overall use of resources? Consequentialism • What will benefit the most and harm the fewest? Deontology • Are there decisions which can be generalised? • Consider the patient as a goal by itself, not only as a means to obtain something else. Virtue ethics. • What is a good doctor in this situation? • What virtues/values are relevant and how are they to be expressed?	What are the options of action in this situation? What arguments are there for and against? Is there a risk stigmatizing? What are the consequences of the actions on both in both short and long term?

The SME model strives to reach a decision or an overall assessment of how the different elements of the value conflict are to be weighed against each other. In the Clinical Ethics Committee of the Region of Southern Denmark, the analysis includes arguments both pro and con in response to the ethical question posed at the beginning of the process.

## Method

When an ethical challenge has been analysed in the Committee, a written case-report of 5–8 pages is made. The case-reports are anonymized, and they serve two purposes. One is to return the Committee’s reflections to the ward it came from to give the clinicians some input, in the form of the case-report, to engage in further joint reflection on their daily practice and possible alternative ways of action related to the case submitted to the Committee. The other purpose is to make it possible also for members of staff at other hospitals, to learn from the case discussion. Therefore the case-reports are made available on the intranet to all clinicians working in mental healthcare in the Region of Southern Denmark.

The case-reports follow the same structure: presentation of the case, the ethical question, facts relevant to the case, the persons involved, ethical analysis (autonomy, doing good/preventing harm, justice), summarising reflections, and response to the ethical question. As is the case in the deliberation process, the case-reports always conclude with arguments both pro and con a concrete way of resolving the ethical challenge. An example of this is example 1. The pros and cons stated to the ethical question asked in example 1 are presented in the result section of this paper. As in example 1, by far the most cases analysed in the Committee are retrospective. A rare example of a prospective case is case number 8. The consequences of the deliberation process in example 8 are also presented in the result section.

The thorough case-reports give good insight into the ethical challenges experienced by clinicians. To extract the overarching topics of the case-reports, we used a qualitative content analysis [18–20], which is a good method to use when straightforward descriptions of phenomena are desired [21].

In the period 2010–2015, 66 case reports were made, of which 55 are included. Eleven case reports have been left out: six are fictitious cases used for training purposes, two are summaries of meetings with little written material available, one case was presented by the patient representative, and two case reports are shaped as a short reply letter only.

First all 55 case-reports were read individually by the first and the second author – a psychiatrist and a philosopher respectively. The reports were read in an open inductive way, focusing on the content of the case-report [22]. The first and the second author read the case-reports “looking for the central conflict of values initiating the ethical challenge” or “filtering out all the less important issues”.

After the individual reading of all case-reports, the first and the second author had several face-to-face meetings in order to reach a consensus about the overarching topics of all the case-reports. Table 1 describes the abductive analytical process of condensing the original ten categories into the three overarching topics.

During the dialogue at these meetings, a consensus was reached about the essence of the central ethical topic or question presented in each case. In this part of the process, the diverse professional and theoretical backgrounds of the two authors became evident; for example in the way that cases concerning the use of coercion were initially categorised differently by the two authors. Through dialogue, they agreed that cases initially categorised by the psychiatrist as cases concerning the use of coercion underneath touched upon other morally relevant topics: What is the extent of the healthcare professionals’ responsibility for potentially harmful actions performed by their patients? When are healthcare professionals obliged to intervene – using coercion – motivated by preventing neglect of care? These cases capture considerations about legitimacy of paternalistic interventions in the life of patients. When is it, for instance, indefensible not to use coercion? Despite the fact that a lot of the case-reports focused on the use of coercion, none of these cases were exclusively about the use of coercion.

A contributory means of identifying and naming the three overarching topics was reflections upon the understanding of ethical difficulties as happening in relations [23, 24] both between individuals but also between individuals and the values existing within an institution or society. Moreover, the analysis process was inspired by studies of the content of case-reports in other parts of the healthcare system [10, 14]. Of special interest was a study presenting an overview of issues brought forward by participants in a moral case deliberation project in two elderly care organizations [25] (Table 2).

**Table 2:** The abductive part of the analytic process

1. Analytic Round	2. Analytic Round	3. Analytic Round: Overarching topics
Use of Coercion (5 Case-reports)	Paternalism or respect of autonomy (13 Case-reports)	Clinicians and their relation to patients and relatives. (19 Case-reports)
Limits of responsibility (8 Case-reports)		
Powerlessness (6 Case-reports)	Futility (6 Case-reports)	
Scarce resources (3 Case-reports)	Scarce resources (3 Case-reports)	Clinicians and institutional aspects of mental healthcare. (23 Case-reports)
Laws (7 Case-reports)	The written and unwritten frame of the mental healthcare institutions (20 Case-reports)	
Guidelines (10 Case-reports)		
Local practice (3 Case-reports)		
Privacy (4 Case-reports)	Privacy (7 Case-reports)	Clinicians and their relation to mental healthcare in a wider social context. (13 Case-reports)
New technologies (3 Case-reports)		
Other values in other institutions (6 Case-reports)	Other values in other institutions (6 Case-reports)	

In the last part of the analysis, the first and the second author decided which case-reports best represented the typical ethical challenges and the diversity within the main topics; see Table 3 in the results section. The diverse professional and educational backgrounds of both the first and the second author as well as the other three members of the research team was used to discuss the analytic process at various stages, to increase the validity by applying researcher triangulation [26].

**Table 3:** Typical ethical challenges within the three main topics

1. Clinicians and their relation to patients and relatives	2. Clinicians and institutional aspects of mental healthcare	3. Clinicians and their relation to mental healthcare in a wider social context
EX 1. Paternalism or respect for the way of life of a psychotic patient EX 2.Powerlessness – when is further treatment futile? EX 3. Involvement relatives: violation of patient autonomy in order to protect the relation with a son EX 4. Solidarity with the patient or respect for a decision made by a colleague	EX 5. Moral distress due to scarce resources EX 6. When an action performed in the best interest of the patient might escalate and be contrary to health legislation EX 7. How to act in the best interest of the patient in a context of conflicting guidelines EX 8. Generalized guidelines are asked for when individual professional judgement is too difficult	EX 9. When easy and quick access to electronic patient records might harm the patient EX 10. To secure privacy of the patient, a psychiatrist refrains from documenting in the electronic patient record EX 11. Is the privacy of the patient violated when the social worker uses Facebook to obtain information? EX 12. When the work of the police is more important than the mental healthcare of the patient

## Results

In this section, the results of the qualitative content analysis will be presented. The results will be presented in three sections, each of which presents one of the three overarching topics: 1. Clinicians and their relation to patients and relatives. 2. Clinicians and institutional aspects of mental healthcare. 3. Clinicians and mental healthcare in a wider social context.

Table 3 shows the three overarching topics with typical examples of ethical challenges or case-reports within each of the main topics. It is important to remember, that the ethical challenges described are most often very complex, and may overlap with other ethical challenges, also among those categorized in another overarching topic. Example 7 is an illustrative example of multiple ethical challenges within one case-report.

The following presentation of the three overarching topics begins with a description of the main normative content of the overarching topic. Afterwards in each main topic, 4 case-reports are presented, illustrating the common ethical challenges within the each overarching topic.

### Clinicians and their relation to patients and relatives.

A characteristic of psychiatric disorders is that the capability of autonomous decision-making may be reduced. This may result in actions that are potentially harmful – both to the health and quality of life of the patient and to other people. When clinicians witness and might prevent potentially harmful actions, they feel responsible. But what are the limits of their responsibility? In these situations, the use of coercion may be the best way to protect the patient’s interests – and a possible way to prevent neglect of care. But the risk of doing harm to the patient, by violating his integrity and his right of self-determination, is always present. The use of coercion may undermine their trust in the mental healthcare system. As a consequence, a patient might be discouraged from turning to the mental healthcare system when needing help in the future. The ethical challenges here centre on balancing doing good/preventing harm to the patient versus respecting the patient’s autonomy.

***Example 1: Paternalism or respect for the way of life of a psychotic patient***

*A is a patient aged between 40 and 50 suffering from schizophrenia and alcohol abuse. A has been chronically ill for many years, hospitalised several times during the last years. Generally, when the condition deteriorates A unassisted contacts the mental hospital. But once A is admitted, A wants to leave. A is then detained and later treated with antipsychotic drugs against A’s will. The treatment with antipsychotic drugs helps reduce A’s psychotic symptoms a little, but almost as important is the reduction of A’s alcohol abuse while admitted. Once treated at the hospital, A is discharged for follow-up treatment at the outpatient clinic. But A turns down their suggestions and initiatives, and for those reasons A is difficult to help. The municipality has refused to find A a group home where A could get care and support. Also, A does not want to live in a group home; instead A is constantly moving to a new apartment because the neighbours complain about A’s behaviour. Sometimes A shouts verbal threats, and some of the neighbours are scared of A. When it all gets too heated up, A vagabonds, also to neighbouring countries. The out-patient clinic wonders whether they are giving A the best possible treatment. Is A suffering unnecessarily? The Committee reflects on respect for the patient and A’s way of living. Is the patient’s strategy of vagabonding, alcohol abuse and finally non-compulsory admission to the mental hospital the best possible way for A to live a tolerable life, or is the situation a result of longstanding neglect of care by the healthcare system?*

*The ethical question in the case-report is: Are clinicians in the out-patient clinic providing the best treatment for the patient?*

- *Yes, because A is capable of seeking help from the mental hospital if A’s condition deteriorates.*
- *Yes, because although clinicians find A’s way of coping harmful to his health, A’s autonomy must be respected.*
- *Yes, because A has never done any physical harm.*
- *No, because A has psychotic symptoms, and therefore A is in no condition to make autonomous decisions.*
- *No, because A is a vulnerable person who needs help and the clinicians may be held liable for neglect of care if they refrain from treatment – if necessary they must use compulsory admission.*
- *No, because A neighbours need protection against his threatening behaviour.*

This case – as many other cases – revolves implicitly around the possibility of using coercion. At the same time, the clinicians are deliberating on the possible harmful consequences of both using and refraining from using coercion.

Sometimes clinicians are faced with patients afflicted in a serious way by mental illness and miserable social circumstances. Unavoidably they develop a relation to the patients. They feel responsible for the patients and the possibilities the patients are given to live a life they find tolerable, and sometimes continued treatment may seem futile. In many cases, a sense of powerlessness and despair is described when all the clinicians’ efforts are failing in easing, relieving or treating the patient. The clinicians are afraid of contributing to neglect of care.

***Example 2: Powerlessness – when is further treatment futile?***

*B is suffering from a severe personality disorder and has been admitted to the locked psychiatric ward for quite a while. B’s behaviour is quite dangerous, and the clinicians have prevented B from suicide several times. On a number of occasions, B has been physically restrained. Some of the clinicians are still on sick leave due to B’s violent actions. Also the fellow patients are affected by B’s behaviour. B has been compulsorily admitted to the department several times by the police because they evaluate B’s actions to be dangerous, both to B and to others. Professionally, the clinicians are of the opinion that continued admission to their department does not constitute good treatment. But B refuses admission to the out-patient clinic. B also refuses to live in a group home. The clinicians do not know what to do. B is suffering and doing a lot of harm, but the clinicians are unable to help B. If they dismiss B from the department, B risks dying as a consequence of B’s own dangerous behaviour. The Committee reflects on the feeling of despair and powerlessness felt by the clinicians. Where is the limit of their responsibility? What are the responsibilities of the patient, who is mentally ill but seldom psychotic?*

The clinicians are caught in a vicious circle, as they have a relation to this suffering patient. They feel obliged to help and to prevent neglect of care. Together with example 9, this case illustrates that sometimes it may not be possible to help. Or the damage done by trying to help might outweigh the benefits. But when is it all right to conclude that all treatment possibilities have been exhausted? When is it best to conclude that for the time being treatment is futile? Is it possible to reverse such a decision?

Sometimes the involvement of relatives or other third parties makes the cases even more difficult.

***Example 3: Involvement of relatives: violation of patient autonomy in order to protect the relation with a son***

*C is a patient aged between 45 and 55 suffering from schizophrenia. C is committed to a mental hospital because C is psychotic suffering from persecutory delusions. C has an adult child D. C is sure that D is about to be killed by a terrorist attack because of C. C is also bothering the dentist, asking for consultations because of dental pain, which C believes to be caused by poisoning. The clinicians are contacted by D. D is troubled by frequent telephone calls from C. If D turns off the phone or fails to pick it up, C calls instead a friend of D or D’s family-in-law. D has in numerous years been safeguarding and helping C, but now D is asking the department to prevent the frequent telephone calls from C. According to the rules of the department, the clinicians are legally in a position to confiscate the patient’s phone. But the patient is – although on psychotic grounds – worried about D. So confiscation might most likely lead to an increased use of sedatives and possibly psychical restraint. Is the harm done to C worth the effort of trying to save the important relation to D? The Committee wonders whether the guidelines of the institution concerning reducing the use of physical restraint may have an influence on the decision of the health-care professionals in this situation.*

The next case raises an important question about the responsibility of clinicians when they experience actions performed by colleagues, which are questionable or criticisable. Out of solidarity with the patient, when and how are clinicians obliged to intervene? And when are they bound by loyalty to defend the actions of a colleague or the mental healthcare institution?

***Example 4: Solidarity with the patient or respect for a decision made by a colleague***

*A nurse, with years of experience from an outpatient clinic, serves as a substitute in a locked psychiatric ward during a period of staff shortage. Together with a colleague from the locked ward, she is called to perform an obligatory inspection of a patient in physical restraint. The patient needs to go to the bathroom. When entering the room, it immediately comes to the attention of the colleague that the leather belt used for physical restraint is far too slack. The colleague walks straight up to the bed and tightens the leather belt. But in the situation, she is disregarding the need of the patient to go to the bathroom. In the view of the nurse, the colleague talks to the patient in a harsh way. The nurse is feeling uncomfortable about the tone of voice used by the colleague. The situation intensifies, and as a result the patient gives up wishing to go the bathroom. The nurse is stunned by the situation, and her own lack of intervention. The Committee reflects on the course of the action of the colleague. The environment in a locked ward may give rise to an increased focus on security. But it still does not justify violation of the integrity of a patient already in a vulnerable and powerless situation. They discuss the challenge of the nurse balancing her feeling of solidarity with the patient against her feeling of loyalty to the colleague, who is a permanent staff member in the locked ward.*

### Clinicians and institutional aspects of mental healthcare

What about the institutional conditions under which clinicians are expected to treat and care for their patients? Ethical challenges may arise when clinicians must *apply and balance various general conditions and rules in specific situations* concerning an individual patient. Sometimes they experience the conditions of care as a barrier to treating patients in the best possible way. The mental healthcare institutions are seen to be responsible for the conditions of care, even though these conditions may be a result of many different elements – some of them beyond the control of the mental healthcare institutions, such as legislation, allocation of financial resources or formulation of national clinical guidelines. Still, mental healthcare institutions are distinct organisations in society, with specific purposes and responsibilities. A mental healthcare institution creates the organisational framework around the daily work and is responsible for local management of wards and clinics. On a day-to-day basis, the mental healthcare institutions provide the main operating conditions for the clinicians, and thus influence the quality of care. Clinicians are expected to be loyal to the mental healthcare institutions and the conditions of care – for example physical conditions, but also written policies and guidelines, routines, budgets, and more informal local practices.

Over the last decades, there has been a decrease in the number of beds in mental healthcare. At the same time, there has been rise in the number of patients in forensic psychiatry. In case of an exacerbation of mental illness in this group, and considering the risk of renewed criminal activity, these patients must be admitted to a locked psychiatric ward. As a result of an overload of patients in the wards of forensic psychiatry, the general psychiatric hospitals receive increasing numbers of patients who should have been admitted to forensic psychiatry.

***Example 5: Moral distress due to scarce resources***

*In a locked ward in a general psychiatric hospital, the clinicians have experienced that three patients from forensic psychiatry have caused a serious change in the environment. They are aggressive in speech and behaviour. One patient in particular has made it necessary to call the police to assist the clinicians several times. Another patient tricks a fellow patient into giving away personal belongings. The clinicians experience the situation as getting out of hand. They know staff levels are higher in forensic wards, and that the staff is better skilled to deal with this group of patients. In this situation, the clinicians feel that the three patients are disrupting the ward, but they are afraid of stigmatising them. The clinicians feel unable to treat both the patients in the general psychiatric hospital and the three patients from the forensic ward in a good way. The Committee reflects on the conditions of care that the clinicians have to work under. The clinicians feel unable to meet their duty to help their patients, and that is felt as a violation of their professional ethical standards.*

The clinicians are torn between being loyal to their “non-forensic” patients and meeting their responsibility to help them – and at the same time being loyal to the mental healthcare institution and the decision to admit patients from forensic psychiatry to a general psychiatric hospital ward, in order to save money.

Health legislation and written policies and guidelines also form part of the conditions of care, because they govern and direct the actions of the clinicians. Ethical challenges arise when the clinicians assess a specific clinical situation and find a way to act that differs from the one described in the guidelines. They may experience that they are at risk of being criminalised for not following the guidelines. Laws or guidelines might be seen as an obstruction to choosing the best possible action. Sometimes the guidelines are self-contradictory and difficult to convert into specific action. In other situations, clinicians may find an individual judgement so difficult to make that they ask for a generalised practice – and more guidelines.

***Example 6: When an action performed in the best interest of the patient might escalate and be contrary to health legislation***

*A clinician F employed in an out-patient clinic of child and adolescent psychiatry is treating E. E is a child with a hyperkinetic conduct disorder and Aspergers syndrome, and aged between 9 and 13. The behavior of E is socially unacceptable. That is a part of the reason why E is admitted to the outpatient clinic. One day E is entering the neighboring out-patient clinic. The waiting room is full of people. E enters and takes a cup of coffee. Then E walks around sprinkling coffee at the people in the waiting room. F discovers the incident, and tries to talk E into stopping. But E goes on, and F is in doubt what to do. Professionally the F is encouraged to use a playful physical intervention, motivated by the fact, that the E needs clear correction of his behavior. But at the same time F knows, that the situation might escalate and get out of hand. F also knows that it is prohibited to use any kind of restraint in an outpatient clinic. F feels that the law prevents F from doing, what F professionally finds is the best action to correct E’s behavior, and protect the people in the waiting room. The Committee reflects on the law made to protect E and E’s right of self-determination. Relocating E using a physical intervention might violate E’s integrity and autonomy and harm E in the short run. In contrast the law may deprive E the good of learning more socially acceptable behavior. Is the harm done proportional to the good gained in the long run? The clinician is divided between the professional assessment of E’s best interest - and acting in loyalty with the law. The clinician is afraid of being criminalized. But what alternative possibilities of action are provided both professionally and organizationally to handle situations like this?*

In other situations, clinicians see the guidelines as self-contradictory and preventing them from acting in accordance with their professional judgment.

***Example 7: How to act in the best interest of the patient in a context of conflicting guidelines***

*Patients admitted to the locked psychiatric ward as an emergency are sometimes unable to buy their cigarettes themselves. To what extent is the psychiatric ward responsible for making cigarettes available? One ward was situated a long distance from the kiosk, and therefore had a box containing cigarettes. Patients could borrow cigarettes from the box, on the condition that they refilled the box when discharged. That did not always happen*. *Controversies about the funding of the cigarettes arose, and the arrangement with the cigarette box stopped. The head of the department could not defend providing the patients with cigarettes due to a general guideline stating that smoking in public areas must decrease or stop. Moreover, psychiatric patients have an excess mortality rate from illnesses related to smoking. But clinicians felt that the timing of motivating patients to quit smoking was not right. They experienced a rise in the use of sedatives, and potentially dangerous situations when patients dependent on nicotine for instance tried to steal cigarettes from their fellow patients. The clinicians were concerned about their own and their patients’ safety, and the risk of increasing use of physical restraint; another guideline stated that the use of physical restraint must be reduced. To prevent escalation of a potentially violent situation, a clinician chose to solve a specific situation by offering a patient one of her own cigarettes. The Committee discussed the conflict between two conflicting guidelines. The clinicians found it difficult to convert the good intentions of diverging guidelines into real life actions.*

In other situations, clinicians ask the mental healthcare institutions for a generalised way of action in complex situations.

***Example 8: Generalised guidelines are asked for when individual professional judgement is too difficult***

*In a clinic treating young people suffering from psychotic disorders, the clinicians often meet young women who are pregnant. It is difficult to build up a treatment alliance with them. But at the same time the clinicians feel as a burden the responsibility of the future child. They are aware of their obligation to notify the municipality if they are worried about the wellbeing of the child. But at the same time, they perceive their treatment relation as important for both the young woman and for her unborn child. They are afraid that a notification to the municipality may be seen as an act of disloyalty. The patients may lose all trust in the clinicians, putting the best possible treatment for their severe mental illness at risk. To secure the treatment alliance, the clinicians may prefer not to inform the municipality, unless the reduced ability to take care of the baby is obvious. But this action may deprive patients of the help they are entitled to, leaving the clinicians with a great responsibility. To avoid that the clinicians refrain from notifying the municipality, the team considers introducing a new practice. They want to tell all pregnant women that the clinicians will always inform the municipality. In that way they disclaim their responsibility of assessing each case individually. The Committee reflects on the fact that the clinicians feel they are snitching on patients when they notify the municipality. The Committee also deliberates on the wish of the clinicians to introduce a general guideline motivated by a wish to avoid this difficult individual clinical judgment. The Mental Health Act specifies that every patient must be seen as an individual person. But the clinicians find it so difficult to make this individual judgment that they have asked for a generalised practice. However, to ask for a general practice is to violate the Act, to avoid their own responsibility and pass on the problem to the municipality.*

As a result of the deliberations in the Committee, the clinicians became aware that by generalising all pregnant women with severe mental illness, they were violating the Act and the right of the patient to get an individual evaluation. In the process, the clinicians gained a better understanding of the difficult ethical elements of cases involving pregnant women. As a consequence, they reflected on and became aware of the legitimacy of involving other clinicians more before deciding whether to notify the municipality or not.

### Clinicians and their relation to mental healthcare in a wider social context

The case-reports in this overarching topic illustrate the ethical challenges that clinicians whose primary responsibility is their patients’ mental health may experience when faced with other values and interests in other institutions and society in general. Patients often have important relations to other institutions as well, for instance somatic healthcare departments, the municipality or the police. These institutions may have other interests than the mental health of the patient – for instance to safeguard the wellbeing of a patient’s unborn child, to prevent abuse of social benefits, or to prevent crime. Clinicians may experience ethical challenges when they have to weigh such considerations against providing mental healthcare for their patients.

New technology has entailed new ways of living and new ways of working – important examples being social media and electronic patient records. These new technologies are motivated by values like openness, involvement of patients and quick and easy communication, which may challenge traditional values within mental healthcare like privacy and patient confidentiality.

***Example 9: When easy and quick access to electronic patient records might harm the patient***

*The process of making a diagnosis involves many clinicians working together. They depend on written facts and considerations documented in the hospital medical records. A clinician who was responsible for the initial diagnostic tests and communication with a patient experienced an ethical challenge when the patient read about the diagnostic considerations in her electronic patient record before the clinician had had a chance to communicate the results to the patient herself. The Committee reflected upon the risk of violating the integrity of the patient, increasing the vulnerability of the patient and decreasing the trust in the healthcare system. The committee also discussed the risk of the clinicians censoring themselves when documenting in electronic medical records. Likewise the committee reflected on the potential responsibility of patients not to read their medical record before the content had been communicated to them by a clinician. Open access to medical records requires that patients know how to read the record and understand its multiple uses. If patients are to be asked not to read certain parts of their records, it will require adequate information from clinicians, and possibly also some technological changes limiting access to various parts of the electronic patient record.*

When using the electronic patient record, clinicians experience ethical challenges because they may unintendedly break patient confidentiality and their obligation to protect patient privacy and integrity.

***Example 10: To secure privacy of the patient, a psychiatrist refrains from documenting in the electronic patient record***

*A pregnant woman sees her psychiatrist G on a regularly basis. During a conversation, G reveals concerns regarding her reduced ability to take care of her future child. G also says that a psychiatrist has an obligation to notify the child protection services in the municipality if there are worries about the wellbeing of a child. At present G is not sure about the graveness of the worries. At the end of their meeting, they agree to postpone the subject to a later meeting, and G agrees not to involve the municipality or anyone else at the moment. After the consultation, G documents in the electronic patient record. Among other things, G briefly writes down the concern that the patient may not have sufficient ability to take care of her future child. Later the pregnant woman sees her midwife. During the preparation for the meeting, the midwife reads the electronic patient record. At the consultation, the midwife mentions the worries of G, and agrees that involving the municipality is the right thing to do. When seeing G again, the woman is disappointed and feels her confidentiality has been violated, and that her privacy and integrity have not been protected. G thinks the accusation is unreasonable since G was just doing what G was supposed to do, documenting relevant matters in the medical record. The Committee reflected on the risk of self-censorship by clinicians, and the potentially negative consequences for both patients and clinicians. The patients risk reduced quality of care. If the clinicians refrain from documenting important knowledge, they become vulnerable because they violate the laws and may be accused of negligence – all to secure the confidentiality of their patients. The Committee also reflected on the conflict of protecting the therapeutic relation for benefit the woman* versus *the responsibility of the midwife to safeguard the wellbeing of the unborn child.*

Social media is another element of the surrounding society that poses ethical challenges to clinicians. The information existing on social media is in a way both private and public.

***Example 11: Is the privacy of the patient violated when the social worker uses Facebook to obtain information?***

*H is a psychologist in the process of diagnosing a patient who has consulted H to discuss difficulties that may be interpreted as symptoms of a personality disorder. H is working on gaining the trust of the patient. Then H is contacted by a social worker from the municipality. When asking H to prepare a written statement of the patient’s state of health, the social worker refers to the patient’s Facebook profile. The social worker suggests that there is a disproportion between the level of function presented by the patient in the Facebook profile and in the consultations with the psychologist. Thereby the social worker raises suspicions about the patient – maybe the patient is not as ill as pretends to be. If that is the case, the patient must take part in a work ability testing programme, from which the patient has been exempted because of assumed mental vulnerability. H is dependent on gaining the trust of the patient if H is to succeed in finding the correct diagnosis. Usually H does not look up the Facebook profiles of patients. H thinks that it violates the privacy, integrity and autonomy of her patients. The interaction with the social worker causes a sense of mistrust against the patient to increase even though H does not find it well-founded. That is not in the best interest of the patient. The Committee reflects that the parties involved use the Facebook profile for different purposes. The patient may give a false picture of a good life and a high level of functioning because the patient wants to avoid stigmatisation by friends. The social worker uses it to discover if the patient is cheating the social services. The primary concern of the psychologist is the health of the patient, and the psychologist has doubts about the applicability of Facebook information. The psychologist is balancing the risk of undermining the trust of the patient and the interest of society to make sure that no one gets away with social welfare fraud.*

As described above in examples 10 and 11, and below in example 12, different institutions have different tasks to perform in society. In example 10, the midwife chooses to safeguard the wellbeing of the unborn child – at the expense of the patient. In example 11, the municipality wants to be sure that the patient does not abuse the social welfare system, but by using methods which may undermine the trust that is needed between patient and clinician.

***Example 12: When the work of the police is more important than the mental healthcare of the patient***

*J is a veteran disabled by PTSD. J lives with his family. The family members are all affected by J’s illness. J witnesses a violent assault in front of their apartment. J does not want to give evidence in court, but J is persuaded to do so by the police. The clinicians are aware that an exacerbation of PTSD symptoms may be expected as a consequence of the stress related to giving evidence in court. The Committee describes how the clinicians are torn between actions based on solidarity with their patient, and loyalty to the institutions of society. They have to weigh consideration for their patient and the family against consideration for the victim of the assault, and the common good of punishing and preventing crime.*

In this last example, the clinicians need to balance different considerations. Their primary responsibility is the health of their patient. The family is also at risk if the mental health problems of the veteran exacerbate. But is this consideration so important that the clinicians should support the veteran in refusing to give evidence in court? The primary task of the police is to fight crime, and to make sure that criminal acts are punished according to the law. On what grounds are clinicians to inform and advise the veteran in this situation? And is J at all in a state to make good decisions concerning both J as an individual and as a member of a family?

## Discussion

The research available often uses a more theoretical basis to classify the ethical content of specific cases. An example is Reiter-Theil et al. using the “Encyclopaedia of Bioethics” [10]. One consequence of this approach is that ethical challenges are often labelled or tagged by only one notification, such as “coercion”, “care management” and “treatment plan evaluation”.

The case reports used in this study are extensive, each about 5–8 pages long. Analytically, the extensive amount of text makes it more difficult to pinpoint the central conflict of values initiating the ethical challenge – or filtering out all the less important issues. The extensiveness adds a lot of complexity to the case reports and an element of entanglement. On the other hand, the strength of the study is the detailed descriptions of ethical challenges and how different considerations, perspectives and conflicts of values are present in a complex and entangled way. A limitation of the study is that it does not add new knowledge to the existing research, as this research tends to mostly focus on a more quantitative description of the frequency of specific ethical challenges.

When reading the case-reports, with the aim of getting insight into the ethical challenges experienced by clinicians in mental healthcare, it is important to have in mind, the specificity of this Committee geographically covering a large area. Of course the case-reports gives important insight into “real life” ethical challenges– but there is a risk, that a lot of ethical challenging situations experienced by clinicians are not captured by the Committee.

It is striking that only one of the cases in this study deals with the issue of disagreement among professionals, or, as in case number 4, a nurse observing what she experiences as a criticisable action performed by a colleague at the expense of the patient. The reason might be, as quoted before, *“a tendency within medical culture to evade conflicts and “outsiders” – meaning persons who are not members of the local medical community in the ward or department”* [27]. In their article, Pedersen et al. have interviewed members of nine Norwegian Clinical Ethics Committees, and they quote a committee member with ample clinical experience:

*So, I guess it is difficult because we do not have a culture for sharing difficult cases outside our own safe little professional community. We do not have a tradition for it. And we tend to think that it either violates confidentiality or that by doing so, one puts one’s own head on the block, or the head of a colleague, that one is disloyal* [27]*.*

For the individual patient, it is vital to be treated with respect and dignity, irrespective of whether he or she is subjected to the use of coercion or not. Maybe, as the quote suggests, the medical culture is a serious barrier for the Committees to deal with these controversial and difficult ethical challenges. To address these important ethical challenges, more locally organized ethical services as moral case deliberation or ethical reflection groups could be a better choice.

*On the other hand, the leader of one committee thought that the committee could be a forum for dealing with serious criticism, criticism which may otherwise either be kept back or end up in the media* [27].

Perhaps clinicians use different kinds of ethic support services for various kinds of ethical challenges? This proposition may be supported by the results of this project, which found more case-reports related to clinicians and their relation to mental healthcare in a wider social context and institutional aspects of mental healthcare. Maybe the clinicians use the Clinical Ethics Committee as a communication channel – a chance to voice the ethical challenges they experience as originating from the institution or the wider social context – in the hope of change?

These reflections emphasise that generalisations about any overall or full picture of ethical challenges found in mental healthcare should be made with great care. Also, in this study the perspectives of patients and relatives were not included. However, this article is in line with other articles [6, 12, 28] that conclude that mental healthcare is a complicated ‘moral enterprise’, both concerning the use of coercion but also in numerous other situations in daily clinical practice. Therefore there is a need for ongoing attention to and awareness of the ethical elements in decision-making processes in the daily clinical practice of mental healthcare.

## Conclusion

The purpose of this article was to give more insight into what ethical challenges clinicians in mental healthcare experience and discuss with a Clinical Ethics Committee in psychiatry. A qualitative content analysis was made of 55 case-reports. Reflections on the understanding of ethical difficulties as happening in relations contributed to identifying and dividing the ethical challenges or the case-reports into three overarching topics. The three overarching topics and the main normative content of each topic are:

1. Clinicians and their relation to patients and relatives; in this overarching topic, the ethical challenges, in different ways, are about the limits of the duty to help.
2. Clinicians and institutional aspects of mental healthcare; described in this overarching topic are the ethical challenges experienced by clinicians when applying and balancing various general rules in specific situations.
3. Clinicians and mental healthcare in a wider social context; in this overarching topic the ethical challenges concerns the social context of society, often prioritizing other values then mental healthcare of patients.

To define the overarching topics and the diversity within each topic, 12 illustrative case-reports are described. It was decided to give “thick descriptions” [29] of each case, including the interests, values and arguments involved in the specific case. Through these “thick descriptions”, a deeper understanding of the complexity and entanglement of different concerns and values emerged.

## Abbreviation

SME: “Senter for Medisinsk Etikk” in Norwegian. In English; Centre for Medical Ethics
